# Post-acquisition filtering of salt cluster artefacts for LC-MS based human metabolomic studies

**DOI:** 10.1186/s13321-016-0156-0

**Published:** 2016-09-06

**Authors:** A. McMillan, J. B. Renaud, G. B. Gloor, G. Reid, M. W. Sumarah

**Affiliations:** 1 Centre for Human Microbiome and Probiotics, Lawson Health Research Institute, 268 Grosvenor Street, London, ON N6A 4V2 Canada; 2 Department of Microbiology and Immunology, The University of Western Ontario, London, Canada; 3 Agriculture and Agri-Food Canada, 1391 Sandford Street, London, ON N5V 4T3 Canada; 4 Department of Biochemistry, The University of Western Ontario, 1151 Richmond Street, London, ON N6A 5B7 Canada

**Keywords:** LC-MS, Metabolomics, Mass defect, Salt cluster

## Abstract

Liquid chromatography-high resolution mass spectrometry (LC-MS) has emerged as one of the most widely used platforms for untargeted metabolomics due to its unparalleled sensitivity and metabolite coverage. Despite its prevalence of use, the proportion of true metabolites identified in a given experiment compared to background contaminants and ionization-generated artefacts remains poorly understood. Salt clusters are well documented artefacts of electrospray ionization MS, recognized by their characteristically high mass defects (for this work simply generalized as the decimal numbers after the nominal mass). Exploiting this property, we developed a method to identify and remove salt clusters from LC-MS-based human metabolomics data using mass defect filtering. By comparing the complete set of endogenous metabolites in the human metabolome database to actual plasma, urine and stool samples, we demonstrate that up to 28.5 % of detected features are likely salt clusters. These clusters occur irrespective of ionization mode, column type, sweep gas and sample type, but can be easily removed post-acquisition using a set of R functions presented here. Our mass defect filter removes unwanted noise from LC-MS metabolomics datasets, while retaining true metabolites, and requires only a list of *m/z* and retention time values. Reducing the number of features prior to statistical analyses will result in more accurate multivariate modeling and differential feature selection, as well as decreased reporting of unknowns that often constitute the largest proportion of human metabolomics data.

**Electronic supplementary material:**

The online version of this article (doi:10.1186/s13321-016-0156-0) contains supplementary material, which is available to authorized users.

## Findings

Untargeted metabolomics has a wide array of applications, from biomarker discovery, to elucidating disease mechanisms, and characterizing the function of microbial communities. Of all available platforms, liquid chromatography-high resolution mass spectrometry (LC-MS) is capable of detecting the widest range of metabolites. The resulting data from a single untargeted LC-MS experiment contains thousands of “features”, where each represents a unique mass-to-charge ratio (*m/z*) and retention time value. Unlike other ‘omics’ fields, annotation of the complete metabolome is not yet realistic, and therefore efforts to identify features are focused on those selected via robust statistical approaches. A consequence of selective annotation is that the proportion of features originating from true metabolites versus background contamination or ionization-generated artefacts remains unknown.

**Fig. 1:**
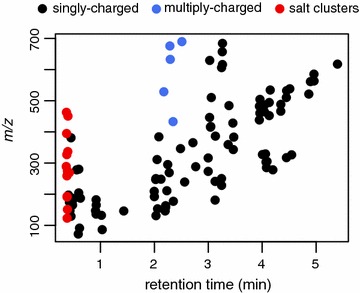
Retention time and *m/z* of plasma metabolites differing significantly between children with severe acute malnutrition and controls (Wilcoxon test, FDR corrected *P* < 0.1, >2 fold change). Salt clusters are shown in *red*, with validated metabolites *colored* according to their charge state. Data was acquired by positive ESI

Our recent work on the plasma metabolome of children with severe acute malnutrition prompted us to address this issue. In this dataset, statistical analysis identified approximately 300 features (positive and negative mode combined) which met our pre-defined *P* value and fold change cut-offs (Wilcoxon test, false discovery rate (FDR) corrected *P* < 0.1, >2 fold change, see Additional file 1: Table S1). However, upon further investigation, we noted that a large proportion of significant features were not endogenous metabolites, but rather salt clusters composed of different combinations of potassium and/or sodium, with chloride and/or formate anions. Although most of these clusters eluted early in the void volume, their retention times overlapped with a number of metabolites of interest, indicating retention time alone is not a suitable filter to remove these artefacts (Fig. 1). The composition of these clusters are not consistent across datasets, and therefore they cannot be removed based on *m/z* alone (data not shown).

Electrospray ionization is known to generate non-covalent complexes, including salt clusters, which can occur irrespective of the extraction method, solvent, chromatography column or MS platform used [1–4]. These clusters are derived from compounds present in the LC buffer and/or compounds present in the sample itself, with the most commonly observed consisting of combinations of small cations such as Na^+^ and/or NH_4_^+^ with chlorides and/or small organic acids, such as formate (HCOO^−^) and acetate (H_3_CCOO^−^) [1]. Salt clusters have *m/z* values with characteristically high mass defects. The exact meaning of mass defect at it pertains to mass spectrometry analysis has been reviewed in detail by Sleno et al. [5], however for this work we simply define the mass defect as the decimal numbers after the nominal mass. This is the result of the relatively high ratio of elements such as chlorine (34.96885 Da), sodium (22.98976 Da), potassium (38.96370 Da), and oxygen (15.99491 Da), compared to hydrogen (1.00782 Da) and nitrogen (14.00307 Da).

**Fig. 2:**
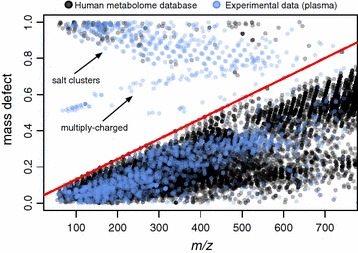
Mass defect as a function of *m/z* for all endogenous and food-derived compounds between 50 and 750 *m/z* in the human metabolome database (hmdb, n = 16,321) compared to features detected experimentally in plasma (n = 2227). The *red line* depicts the theoretical mass defect maxima for compounds containing only C, H, N and/or O (y = 0.00112x + 0.01953). Features above this *line* which are present only in the experimental dataset are likely to be salt clusters or multiply charged as indicated by the *arrows*. Hmdb masses are shown as theoretical [M + H]. Experimental data was acquired by positive ESI, and contains a variety of adducts including but not limited to [M + H], [M + Na], [M + K] and [M + NH_4_], as well as isotopic peaks

To evaluate the occurrence of high mass defect compounds in human metabolism, the mass defect of all endogenous or food-derived metabolites in the human metabolome database (hmdb) [6] were plotted by *m/z* (Fig. 2; Additional file 2: Table S2). Only 0.38 % of endogenous compounds (modelled as [M + H]) fell within the mass defect space occupied by salt clusters, confirming the rarity of human metabolites with such high mass defects. When all common adducts were considered, the percentage of hmdb metabolites in salt cluster space only increased to 3.34 % for positive mode and 1.84 % for negative mode (Additional file 3: Table S3).

Given the ubiquity of salt clusters in LC-MS data [1–4], and their predictable mass defect, we developed a method to identify and remove salt cluster artefacts from untargeted LC-MS data using mass defect filtering. This comprised performing a linear regression of compounds with the highest mass defect in the hmdb (Fig. 2), then modelling C_n_H_n+2_ alkanes, which represent the theoretical maxima mass defect for compounds containing only carbon, hydrogen, oxygen, and/or nitrogen. Both methods yielded the same linear equation (y = 0.00112x + 0.01953). We then applied this equation to experimental datasets and removed feature-s with mass defects greater than our model equation. Compounds containing other elements such as sulfur, phosphate, and iodine were included in this analysis, but were not used in generation of the model equation as they form high mass defect compounds such as sulphates, phosphates and thyroid hormones. Given the small number of hmdb compounds with high mass defects, we also incorporated an “inclusion list” into our model (Additional file 2: Table S2). Features in experimental datasets with the same *m/z* as compounds in this inclusion list (within a pre-set error range) will be retained; ensuring known endogenous compounds are not removed with salt clusters.

**Fig. 3:**
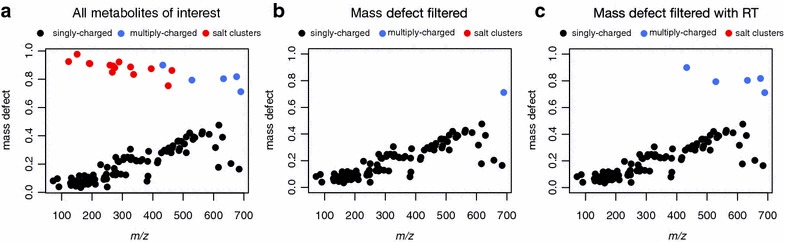
Significant metabolites (Wilcoxon test, FDR corrected P < 0.1, >2 fold change) remaining in the malnutrition dataset after **a** no filter, **b** mass defect filter only, **c** mass defect filter with retention time. Each point represents a single metabolite, colored according to their charge states, with salt clusters shown in *red*. Data was acquired by positive ESI. Singly-charged features encompass a variety of adducts including but not limited to [M + H], [M + Na], [M + K] and [M + NH_4_]

To test the ability of our method to remove artefacts while retaining validated metabolites, we applied this filter to plasma data from the metabolomics study of severe acute malnutrition mentioned previously (Additional file 1: Table S1). Importantly, the metabolites of interest contained multiply-charged peptides, which were not modelled by the hmdb dataset (Fig. 2). Some of these peptides occupied the same mass defect space as the salt clusters, and therefore would be removed from the analysis by our original, ‘mass defect only’ method (Fig. 3a, b). However, using a C18 column, the salt clusters elute in, or shortly after the void volume, while peptides are retained (see Additional file 4: Figure S1). We therefore incorporated retention time into the model as a third variable to further isolate the salt clusters. Incorporation of retention time removed all salt clusters while retaining all identified metabolites of interest, confirming the validity of the method (Fig. 3c).

We next applied the mass defect filter to all features detected in plasma (2227 in positive mode, 1742 in negative mode) to determine the percentage of features in the complete dataset with mass defects corresponding to salt clusters. This analysis revealed a large percentage (15.94 % in positive mode, 28.47 % in negative mode) of total features were likely salt clusters (Table 1).

**Table 1:** Percent data reduction after mass defect filtering alone or in combination with retention time and hmdb inclusion list

Ionization mode	Filter	Features remaining	% Features removed
Positive	None	2227	0.00
	md	1730	22.32
	md + RT	1853	16.79
	md + RT + inclusion	1872	15.94
Negative	None	1742	0.00
	md	1107	36.45
	md + RT	1225	29.68
	md + RT + inclusion	1246	28.47

All features detected in plasma in the malnutrition dataset are shown in both positive and negative ionization mode

*RT* retention time, *md* mass defect

**Fig. 4:**
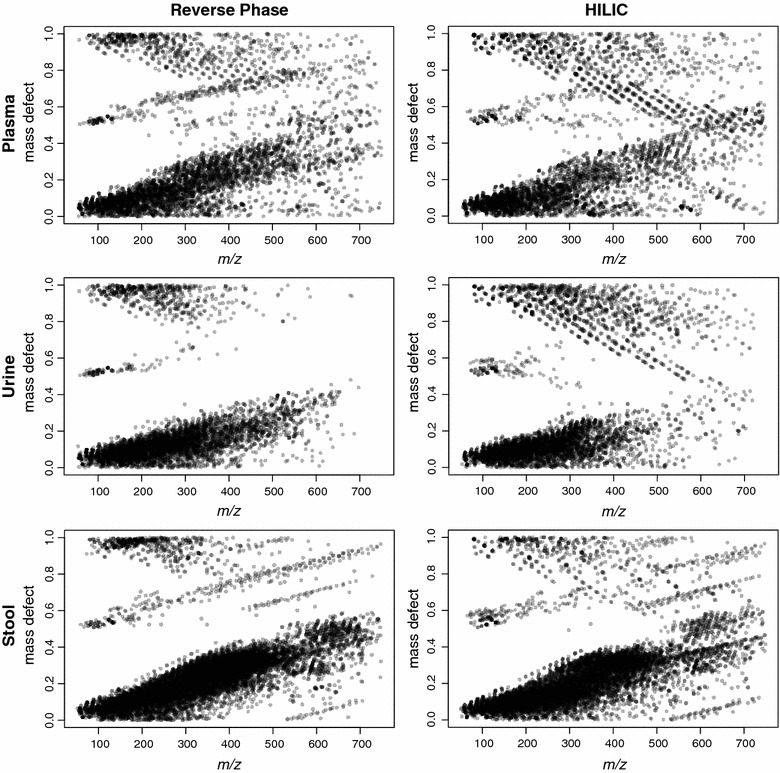
Effect of column type on salt cluster formation in plasma, urine and stool. The *left* and *right panels* display mass defect as a function of *m/z* for the same set of samples run on reverse phase or HILIC columns respectively. Data was acquired by positive ESI

To determine if the proportion of salt clusters could be reduced instrumentally, and if they occurred in other biological matrices, we ran a series of tests comparing the effect of sweep gas and column type (reverse phase or HILIC) on salt cluster formation in a set of three plasma, urine and stool samples (see Additional file 5: Supplementary Methods for details). The sweep gas did not significantly reduce the salt cluster proportion (Additional file 6: Table S4), although fewer features were detected overall, indicating lower sensitivity with this method. Surprisingly, the use of HILIC columns consistently increased the proportion of salt clusters (Fig. 4; Additional file 6: Table S4), perhaps due to less ion suppression at later retention times where these salt clusters elute [7, 8] (Additional file 4: Figure S1).

Although we identified salt clusters in all sample types, they consistently occurred at a lower proportion in stool and urine compared to plasma. The use of K_2_EDTA tubes for blood collection in our study may be responsible for this observation. Barri et al. [9] demonstrated that plasma collected with EDTA tubes had a significantly higher number of potassium clusters compared to heparin tubes, while citrate tubes resulted in more sodium clusters. These results indicate that salt cluster removal is applicable to stool, urine and plasma, but is particularly important for plasma collected with tubes containing salt-based anticoagulants.

It is worth noting that our method was designed for studies pertaining to human physiology, and therefore synthetic compounds were not included in the analysis. Synthetic compounds such as drugs and pesticides, which are more likely to contain halogens [10, 11], can occupy the salt cluster mass defect space. Investigators concerned with these types of molecules may therefore wish to incorporate these masses in the inclusion list.

The advantage of removing artefacts prior to annotation and statistical analyses is three-fold. Firstly, removing a large number of unknown features will allow for more complete annotation of the metabolome, and decreased reporting of false positives. Secondly, feature reduction may change the relationship between samples as determined by multivariate modelling methods such as principal component analysis. Most importantly, removing hundreds of features will affect the distribution of *P* values generated from univariate analyses. This has important implications for multiple testing corrections, such as the false discovery rate (FDR) and Bonferroni adjustment, which rely on this distribution (FDR), or on the total number of features compared (Bonferroni) for *P* value adjustment [12, 13].

In conclusion, we propose a method to filter out salt cluster artefacts in untargeted LC-MS data using mass defect and retention time. This filter can be easily applied to processed data using a set of R functions, and requires only a list of detected *m/z* and retention time values. The code for these analyses as well as example datasets are freely available at (https://github.com/amcmil/mz_defect_filter).

## Availability

The R code and example data sets are freely available at (https://github.com/amcmil/mz_defect_filter).

## Additional files

10.1186/s13321-016-0156-0 LC-MS features detected in plasma from a study of children with malnutrition.
10.1186/s13321-016-0156-0 Mass defect of all endogenous and food-derived metabolites in the human metabolome database.
10.1186/s13321-016-0156-0 Number of metabolites in the human metabolome database with high mass defects when expressed as common adducts.
10.1186/s13321-016-0156-0 Retention time distribution of ions in salt cluster space.
10.1186/s13321-016-0156-0 Supplemental methods.
10.1186/s13321-016-0156-0 Effect of sweep gas and column type on salt cluster formation.

## Abbreviations

hmdb: human metabolome database
LC-MS: liquid chromatography-mass spectrometry
*m/z*: mass-to-charge ratio
FDR: false discovery rate
