# Supplementary material for: Post-acquisition filtering of salt cluster artefacts for LC-MS based human metabolomic studies
Source: J Cheminform. 2016 Sep 6;8(1):44. doi: 10.1186/s13321-016-0156-0 (PMC5013591; doi:10.1186/s13321-016-0156-0)
Supplement: Supplementary file 3 — 10.1186/s13321-016-0156-0 Number of metabolites in the human metabolome database with high mass defects when expressed as common adducts. [file 13321_2016_156_MOESM3_ESM.docx]

| **ionization mode** | **adduct** | **N high md metabolites** | **% unique metabolites with any high md adduct** |
| --- | --- | --- | --- |
| **positive** | [M+H] | 147 |  |
|  | [M+Na] | 295 | 3.34 |
|  | [M+K] | 883 |  |
|  | [M+NH4] | 147 |  |
| **negative** | [M-H] | 263 |  |
|  | [M+FA-H] | 213 | 1.84 |
|  | [M+Cl-] | 695 |  |

**Table S3**: Number (N) of metabolites in the human metabolome database with high mass defects (md) when expressed as common adducts. The far right column indicates the percentage of unique metabolites with any high md adduct as determined by mass defect filterering.
