# Supplementary material for: Post-acquisition filtering of salt cluster artefacts for LC-MS based human metabolomic studies
Source: J Cheminform. 2016 Sep 6;8(1):44. doi: 10.1186/s13321-016-0156-0 (PMC5013591; doi:10.1186/s13321-016-0156-0)
Supplement: Supplementary file 4 — 10.1186/s13321-016-0156-0 Retention time distribution of ions in salt cluster space. [file 13321_2016_156_MOESM4_ESM.pdf]

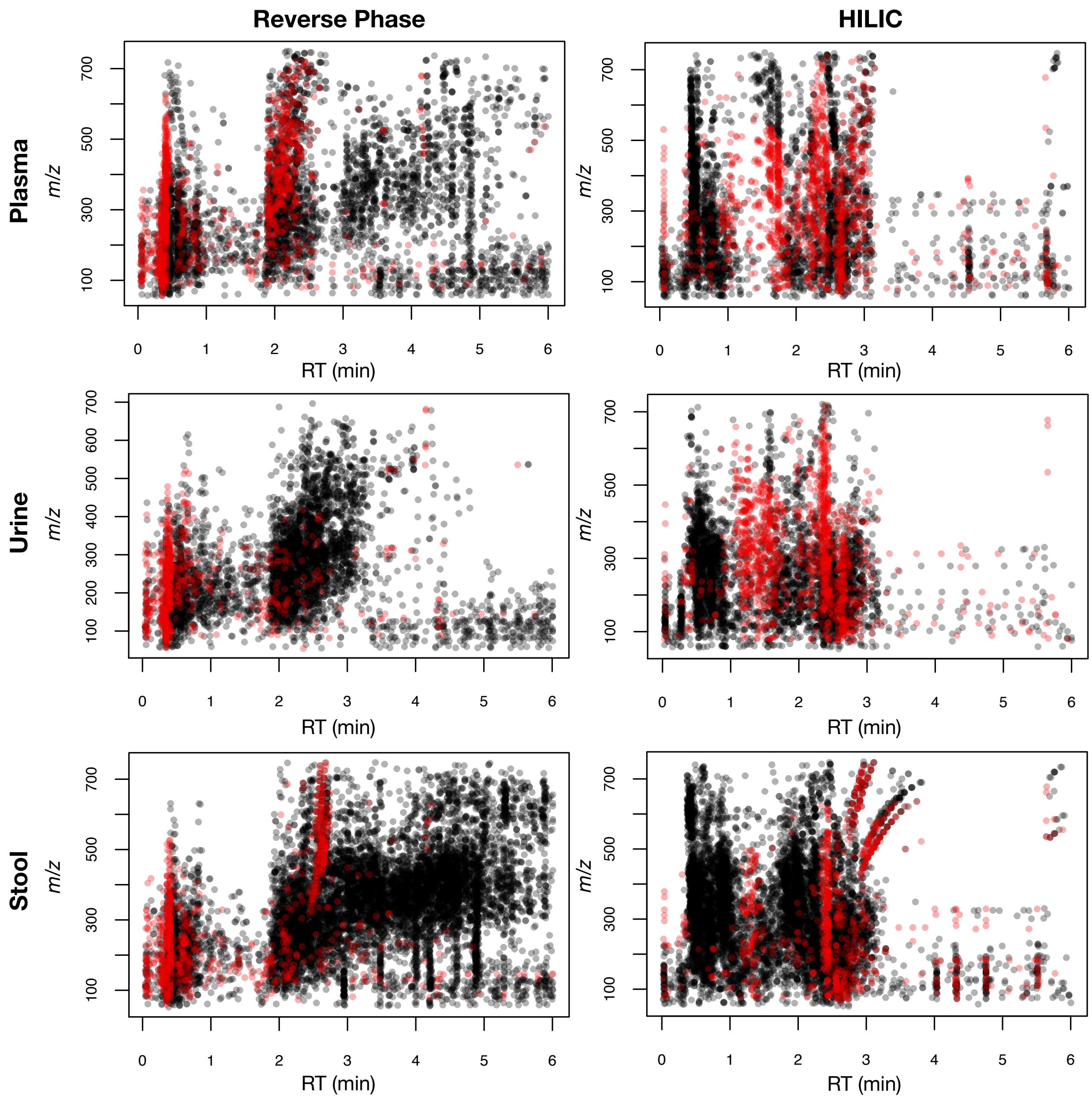

Figure S1. Retention time distribution of ions in salt cluster space. The left and right panels display retention time as a function of *m/z* for the same set of samples run on reverse phase or HILIC columns respectively. Red points indicate high mass defect ions identified by mass defect filtering in combination with the hmdb inclusion list. Retention time was not included in the model due to the different elution times of clusters in reverse phase compared to HILIC. Data was acquired by positive ESI.
