# Supplementary material for: Post-acquisition filtering of salt cluster artefacts for LC-MS based human metabolomic studies
Source: J Cheminform. 2016 Sep 6;8(1):44. doi: 10.1186/s13321-016-0156-0 (PMC5013591; doi:10.1186/s13321-016-0156-0)
Supplement: Supplementary file 5 — 10.1186/s13321-016-0156-0 Supplemental methods. [file 13321_2016_156_MOESM5_ESM.docx]

**Supplemental Methods**

**Metabolite extraction**

For stool, approximately 250 mg of wet sample was lyophilized overnight. After drying, 40 mg was weighed into microcentrifuge tubes and extracted with 8:2 methanol:H_2_O to a final concentration of 40 mg/mL. Samples were then vortexed for 30 sec, followed by centrifugation for 15 min at 10 000 rpm. Supernatant was then transferred to LC-MS vials for with micro-inserts for analysis.

Metabolites were extracted from plasma according to the methods of Dunn *et al*, 2011 (1). Briefly, plasma samples were thawed on ice for 30 min. Once thawed, 805 μL of 8:2 methanol:H_2_O was added to 230 μL of plasma to make a 4.5 fold dilution. Samples were vortexed for 15 sec and centrifuged at 15 000 rpm for 15 min to pellet precipitated proteins. 370 μL of supernatant was then transferred to separate vials and dried down for LC-MS using a speedvac with no heat. Samples were then reconstituted with 90 μL ddH_2_O and transferred to LC-MS vials with micro-inserts for analysis.

For urine, 200 μL of sample was extracted with 800 μL 1:9 Acetonitrile:H_2_O as per the methods of Warth *et al.* 2012 (2). After centrifugation, 500 μL of supernatant was transferred to LC-MS vials with micro-inserts for analysis.

**LC-MS analyses**

Samples were analyzed using an Agilent 1290 Infinity HPLC coupled to a Q-Exactive Orbitrap mass spectrometer (Thermo Fisher Scientific, Waltham, USA) with a HESI (heated electrospray ionization) source. For reverse phase HPLC, 2 μL of each sample was injected into a ZORBAX Eclipse plus C18 2.1 x 50mm x 1.8 micron column. Mobile phase (A) consisted of 0.1% formic acid in water and mobile phase (B) consisted of 0.1% formic acid in acetonitrile. The initial composition of 0% (B) was held constant for 30 s and increased to 100% over 3.0 min. Mobile phase B was held at 100% for 2 minutes and returned to 0% over 30s for a total run time of 6 min. For normal phase HPLC, 2 μL of each sample was injected into a ZORBAX RRHD HILIC plus 2.1 x 50mm x 1.8 micron column. Mobile phase (A) consisted of 0.1% formic acid in water and mobile phase (B) consisted of 0.1% formic acid in acetonitrile. The initial composition of 95% (B) was held constant for 30 s and decreased to 5% over 3.0 min. Mobile phase B was held at 5% for 1 minute and returned to 95% over 30s and held for 1 minute for a total run time of 6 min.

Full MS scanning between the ranges of *m/z* 50-750 was performed at 140 000 res–olution. The HESI source was operated under the following conditions: nitrogen flow of 30 and 8 arbitrary units for the sheath and auxiliary gas respectively, probe temperature and capillary temperature of 450 °C and 250 °C respectively and spray voltage of 3.9 kV and 3.5 kV in positive and negative mode respectively. The automatic gain control (AGC) target and maximum injection time were 1e6 and 500 ms respectively. For experiments testing the affect of sweep gas on cluster formation, sweep gas was set to 2 arbitrary units. Blanks of pure methanol were run between every sample to limit carryover. After data acquisition Thermo .RAW files we­re converted to .MZML format and centroided using ProteoWizard (3). Files were then imported into R using the XCMS package (4) for chromatogram alignment and deconvolution. Features were detected with the “xcmsSet” function using the “centWave” method and a ppm tolerance of 1. Prefilter was set to 3-5000, noise 1E5, and signal to noise threshold was set to 5. Due to a lower overall noise and signal in negative mode, noise was set to 1E3 for this mode. Retention time correction was conducted using the “obiwarp” method, grouping included features present in at least one samples, allowable retention time deviation was 5 seconds, and *m/z* width set to 0.015. Areas of features below the signal to noise threshold in the data were integrated using the “fillPeaks” function with default settings.

**References**

1. Dunn WB, Broadhurst D, Begley P, Zelena E, Francis-McIntyre S, Anderson N, et al. Procedures for large-scale metabolic profiling of serum and plasma using gas chromatography and liquid chromatography coupled to mass spectrometry. Nat Protoc. 2011;6(7):1060–83.

2. Warth B, Sulyok M, Fruhmann P, Mikula H, Berthiller F, Schuhmacher R, et al. Development and validation of a rapid multi-biomarker liquid chromatography/tandem mass spectrometry method to assess human exposure to mycotoxins. Rapid Commun Mass Spectrom. 2012 Jul 15;26(13):1533–40.

3. Kessner D, Chambers M, Burke R, Agus D, Mallick P. ProteoWizard: open source software for rapid proteomics tools development. Bioinformatics. 2008;24(21):2534–6.

4. Patti GJ, Tautenhahn R, Siuzdak G. Meta-analysis of untargeted metabolomic data from multiple profiling experiments. Nat Protoc. 2012;7(3):508–16.
